# Design and computational optimization of compliance-matching aortic grafts

**DOI:** 10.3389/fbioe.2023.1179174

**Published:** 2023-06-29

**Authors:** Georgios Rovas, Vasiliki Bikia, Nikolaos Stergiopulos

**Affiliations:** Laboratory of Hemodynamics and Cardiovascular Technology (LHTC), Institute of Bioengineering, Swiss Federal Institute of Technology (EPFL), Lausanne, Switzerland

**Keywords:** distensibility, synthetic, compliance mismatch, stent graft, arterial prosthesis, aortic replacement, FEM FEA, nickel titanium (NiTi-nitinol)

## Abstract

**Introduction:** Synthetic vascular grafts have been widely used in clinical practice for aortic replacement surgery. Despite their high rates of surgical success, they remain significantly less compliant than the native aorta, resulting in a phenomenon called compliance mismatch. This incompatibility of elastic properties may cause serious post-operative complications, including hypertension and myocardial hypertrophy.

**Methods:** To mitigate the risk for these complications, we designed a multi-layer compliance-matching stent-graft, that we optimized computationally using finite element analysis, and subsequently evaluated *in vitro*.

**Results:** We found that our compliance-matching grafts attained the distensibility of healthy human aortas, including those of young adults, thereby significantly exceeding the distensibility of gold-standard grafts. The compliant grafts maintained their properties in a wide range of conditions that are expected after the implantation. Furthermore, the computational model predicted the graft radius with enough accuracy to allow computational optimization to be performed effectively.

**Conclusion:** Compliance-matching grafts may offer a valuable improvement over existing prostheses and they could potentially mitigate the risk for post-operative complications attributed to excessive graft stiffness.

## 1 Introduction

Synthetic vascular grafts are widely used in clinical practice for vascular and cardiac surgery for over 50 years (Leon and Greisler, 2003). One of their most successful applications is the replacement of the thoracic and abdominal aorta and, more recently, the aortic root, either by open surgery or by endovascular repair. Aortic replacement is one of the main courses of treatment for a variety of medical conditions, including aortic aneurysms, dissections, dilatations, Marfan syndrome, and aortic valve diseases. The prevalence of some of these diseases is very high, namely, 1.2%–1.4% for abdominal aortic aneurysms (Lederle et al., 1997; Lederle, 2000), and 0.16%–0.34% for thoracic aortic aneurysms (Itani et al., 2002; Kälsch et al., 2013). Unsurprisingly, the incidence rate of these diseases is increasing due to the continuous aging of the population, the increase of related comorbidities and causal factors, and perhaps due to the increasing frequency of medical imaging of the aorta (Kuzmik et al., 2012). Additionally, aortic aneurysms are consistently ranked as one of the leading causes of death, a fact that could be partly attributed to the lethal nature of aneurysms, that remain undetected until they rupture, often causing fatal consequences (Elefteriades and Rizzo, 2007).

Although there have been major improvements in synthetic grafts for large arteries to facilitate the application and reduce the operation time, little has been done to address their inability to mimic the elastomechanical properties of the native aorta. Elasticity is a major concern with regards to the aorta, because it is its compliance that allows the aorta to act as a buffer to accommodate the pressure oscillations during the cardiac cycle and to propel the blood to the periphery. This function is known as the “Windkessel effect.” When synthetic grafts, that are multiple times stiffer that the native tissue, replace the aorta, the aortic compliance is decreased substantially (Vardoulis et al., 2011). This, in turn, causes the total arterial compliance to decrease, because approximately 60% of the arterial compliance resides in the proximal aorta (Stergiopulos et al., 1999). The first indications of the hemodynamic load that aortic grafts impose on the cardiovascular system came from aortic bypass grafting in dogs (Maeta and Hori, 1985). Moreover, multiple studies have shown the deleterious effect of reduced arterial compliance on the cardiovascular system that leads to the augmentation of systolic and pulse pressure (Ioannou et al., 2003) and, subsequently, to other cardiovascular complications like hypertension, myocardial hypertrophy (Ioannou et al., 2009), reduced coronary flow and even cardiac ischemia (Maeta and Hori, 1985). The effects of reduced systemic compliance, have been excessively studied in the last decades due to their major contribution in the genesis and progression of essential hypertension (Randall et al., 1984; Laurent et al., 2001; Mitchell, 2014). In addition to reduced compliance, some frequent graft complications, such as rupture, migration, endoleak, infection and re-stenosis, could also be partially attributed to the abrupt change of properties between the graft and the artery (Major et al., 2006; Singh et al., 2017; Post et al., 2019).

The difference in elastomechanical properties between the healthy aortic tissue and the aortic grafts is often termed as “compliance mismatch.” The absence of compliant aortic grafts has dissatisfied clinicians and it has led scientists to question the ability of existing grafts to mimic the physiological function of the aorta (Spadaccio et al., 2013). Researchers have proposed a biomimetic graft, composed of multiple polymers with two lumina that could alleviate some of the problems (Singh and Wang, 2014), but the recent scientific work has been mostly directed towards identifying the medical problems attributed to existing grafts (Spadaccio et al., 2016). Notable progress has been made in the field of small-diameter graft development, with implementation of new materials (Li et al., 2015; Nezarati et al., 2015; Singh et al., 2015; Montini-Ballarin et al., 2016) and cellularized biodegradable graft designs (Tiwari et al., 2002; Rashid et al., 2004). However, these solutions are unlikely to work in large arteries, due to the different design requirements. These findings have prompted researchers to characterize long-term, compliant grafts as a “fleeting reality” (Lucereau et al., 2020).

Here, we aimed to design a biomimetic and biocompatible vascular graft that is matching the compliance of healthy human aortas. We optimized the graft’s properties computationally based on finite element method (FEM) simulations. Consequently, we fabricated the graft and measured its properties *in vitro* via simultaneous acquisition of pressure, volume, and radial displacement. Thereby, we validated the FEM simulation and verified that the prosthesis manifests the specified compliance in typical implantation conditions.

## 2 Materials and methods

### 2.1 Compliance-matching graft design

**FIGURE 1:**
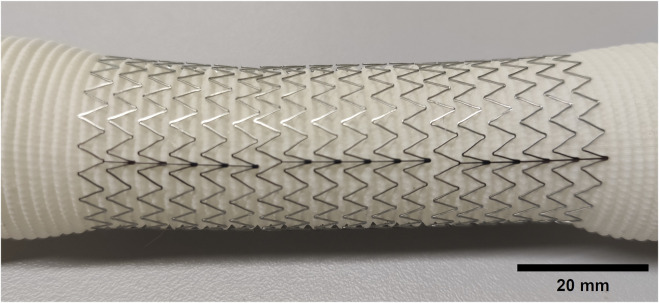
Photograph of a compliance-matching aortic graft.

To design the compliance-matching (CM) graft, we drew inspiration from the natural structure of large elastic arteries. Their medial layer consists of a network of interconnected elastin fibers, stiffer collagen fibers, and smooth muscle cells. In the unloaded state, both the elastin and collagen fibers are undulated and wavy. However, at physiological pressure, the elastin fibers are mostly disentangled, while the collagen fibers are only partially engaged (Wagenseil and Mecham, 2012). This allows arteries to be adequately elastic at normal conditions and to have the necessary strength at higher pressure. We implemented a multi-layer design (Figure 1), similar to the arterial wall structure. As the inner layer we used a stiff woven graft, equivalent to the existing grafts, which have been used in clinical practice for many years. To prevent the stiff graft from bearing load, we compressed it circumferentially with an outer layer, so that it had margin to uncoil before its fibers become engaged. The stiff graft resembled the role of collagen and provided the necessary mechanical strength. As an outer layer, we designed an easily deformable z-shaped stent, which imitated the function of elastin fibers. The outer layer compressed the inner layer and it played a crucial role in controlling the radial expansion and, hence, the compliance of the whole graft.

Importantly, the compliance or equivalently, the deformability of a construction does not depend solely on its material, but also on its shape and structure. It may seem counterintuitive that the metallic stents are more compliant that the woven graft, but it is a feature that could be achieved by appropriate design. A thin metallic tube, for example, could be more compliant that a thicker plastic one, even if the elastic modulus of the metal is much higher than that of the plastic. This can be easily proven by using the Moens-Korteweg equation: $PWV=\sqrt{\left(E_{inc}h\right)/\left(2r\rho \right)}$ , where PWV is the pulse wave velocity, a measure of vessel compliance, $E_{inc}$ is the incremental elastic modulus, h is the wall thickness, r is the vessel radius and ρ is the density of blood. In this equation, we can also distinguish the material stiffness, which is defined by $E_{inc}$ , from the structural stiffness, which is defined by the product $E_{inc}h$ (Butlin et al., 2020). In our design, we utilized a shape that turned the radial expansion of the graft into a bending-dominated behavior at the metallic stents (the axes of the bending moments were perpendicular to the axis of the tubular graft). Additionally, we took advantage of the stress concentration at the corners where the struts meet, and the ability to create very thin struts, to create a highly compliant stent.

### 2.2 Materials and fabrication

The inner layer consisted of a pleated, woven, commercial graft (Gelweave, Terumo Corporation, Tokyo, Japan), which was made of polyethylene terephthalate (PET) and was impregnated with gelatin. The inner layer was one of the current gold-standard aortic grafts, which are recognized for their mechanical strength, ease of manipulation and their long-term size retention (Troost et al., 2009). The nominal diameter of the inner layers was 16–30 mm. We manufactured the stents from laser-cut and electropolished nickel titanium (Nitinol) tubes, that were shape-set to the appropriate diameter. We selected this material for its biocompatibility and its excellent, for the present application, mechanical properties. The stents were designed so that at body temperature the material would be between the austenite finish temperature (A_f_) and the martensite deformation temperature (M_d_), resulting in superelastic elastic behavior. The stent diameter was smaller than the diameter of the PET graft (inner layer), to achieve the necessary compression. The stents were inserted over the inner layer with a custom mandrel and were secured using 2-0 non-absorbable silk sutures with two suture points per stent. We fabricated grafts of three diameters to verify that the design approach could work for grafts of varying sizes.

### 2.3 Computational model

**FIGURE 2:**
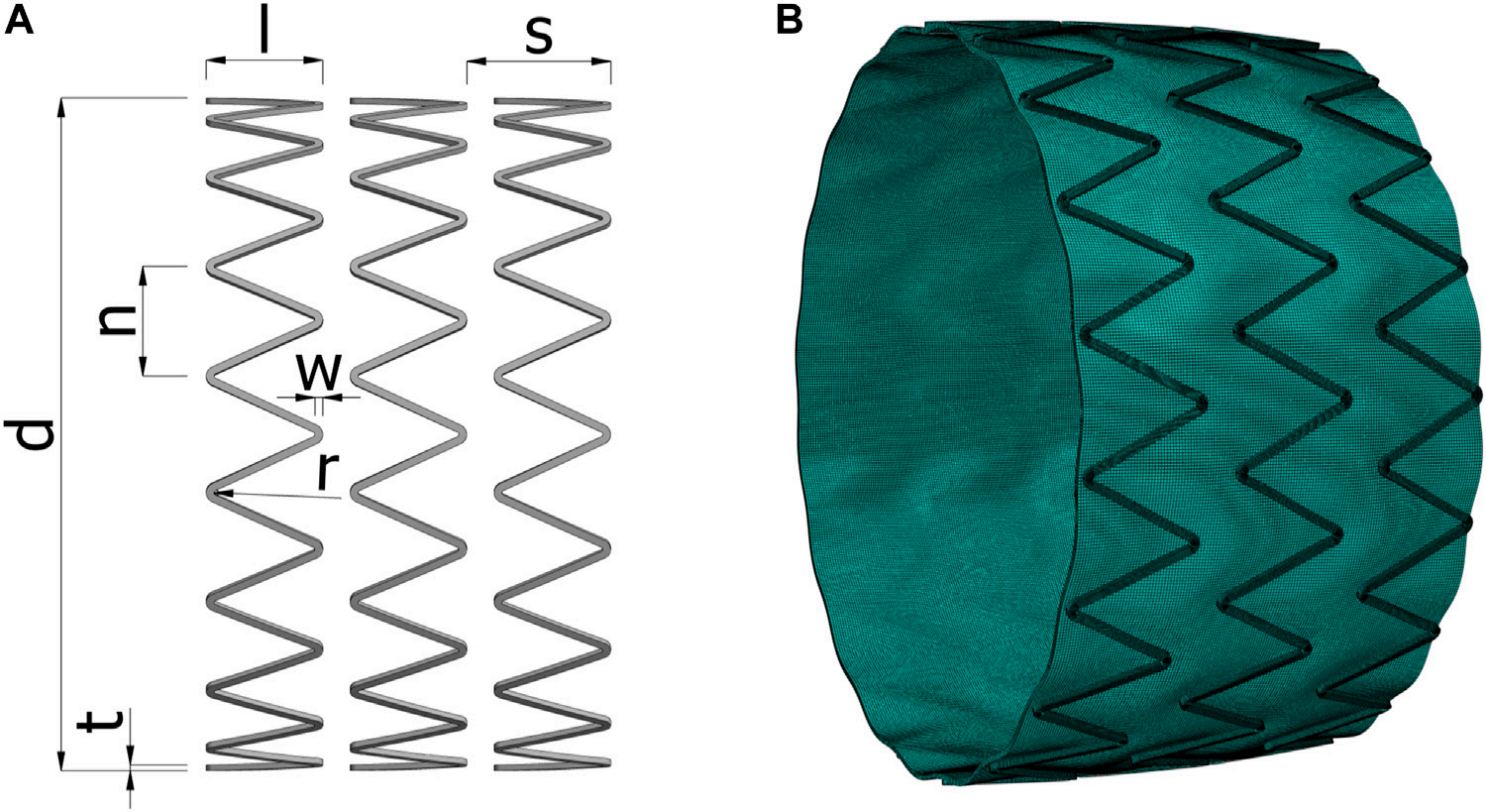
**(A)** The geometrical parameters of the stent. **(B)** Example of the deformed mesh of an algorithmically generated model at internal pressure of 120 mmHg, solved with the finite element simulation. d, stent diameter; l, strut length; s, stent spacing; n, circumferential step; w, strut width; r, radius of curvature; t, strut thickness.

We created a detailed FEM simulation of the composite graft, aiming to predict its behavior and specifically, its area compliance. We designed a generalized stent model completely parametrically, so that we can control the design with a small set of parameters, including the stent diameter, strut length, stent spacing, circumferential step, strut width, radius of curvature, and strut thickness (Figure 2A). Then, we developed a custom program that could read the parametric design and build the stent-graft assembly automatically, while maintaining the same meshing options, loads and boundary conditions, regardless of the design. We implemented the program for the algorithmic preparation of FEM models in Python (Python Software Foundation, DE, United States). We modelled a length of three consecutive stent rings to minimize boundary effects (Figure 2B). Based on a mesh independence study, we meshed the strut cross section with an 8 × 8 element grid of hexahedral second-order elements with incompatible modes, and the graft with a grid of 4 hexahedral elements with hybrid formulation and incompatible modes, equally distributed along the graft’s thickness. The remaining mesh elements dimensions were maintained the same between different designs. In such a manner, we ensured that the solution will be independent of the assembly and the meshing procedure, across designs of different dimensions.

We imposed the same FEM modelling parameters to all the designs. The PET graft was modelled as an isotropic, hyperelastic, third-order Ogden material (Ogden and Hill, 1972) with near incompressible behavior. For the nitinol graft, we used a custom, thermomechanical, superelastic material model, with no plasticity (Auricchio et al., 1997). We estimated the parameters of the PET and nitinol material models from experimental measurements by parameter fitting and direct derivation, respectively. Large deformation analysis was necessary, due to the bending-dominated nature of the problem. We modelled the contact between the two layers on the normal direction as hard contact, to avoid penetration of one surface into the other, using the penalty enforcement method, and on the tangential direction as isotropic friction, again with penalty enforcement. The simulation was divided in different stages, that resembled the stages of the assembly and evaluation process, namely, the compression of the graft with a mandrel, the release of the compressed graft by allowing contact with the stents, and the application of internal pressure. We imposed symmetric boundary conditions on all axial boundaries. The imposed loads varied according to the simulation stage, and they included the compressive load imposed on the inner layer and the intraluminal pressure imposed on the inner surface of the graft that linearly increased up to 200 mmHg. The temperature was set to body temperature throughout the simulation. The simulation was conducted in Abaqus (Dassault Systèmes, Vélizy-Villacoublay, France) using the explicit solver. Additional information regarding the simulation parameters can be found in Supplementary Material.

### 2.4 Optimization

Initially, we performed a sensitivity analysis to determine the subgroup of stent design parameters that had a major effect on the compliance of the graft. These parameters consisted of the stent dimensions and the thermal properties of the material. We excluded parameters that we would not be able to control during the manufacturing. We selected the six parameters with the largest main effects on compliance and we created a 6-factor Box-Behnken design (Box and Behnken, 1960) to calculate the response surface. We chose this type of design to get an accurate estimation of the gradient of the response surface, while minimizing the number of computational models that would have to be solved. We calculated the response surface by fitting a quadratic model to the simulation results of each design. Based on the response surface, we implemented a gradient descent methodology, to translate and shrink the Box-Behnken design in the 6-dimensional space, and hence define the Box-Behnken design points of the next optimization cycle. As the objective function we used the difference between the distensibility of the grafts and the mean distensibility of human aortas. This process was continued until the change in the mean compliance of consequent optimization cycles was less than 1%. We repeated the procedure from different starting points, and we selected the stent design with the optimal compliance. For the optimization of grafts with different diameters or materials, the optimization process had to be restarted from the beginning. The computational models were solved on high performance computing clusters, while the parallelization was achieved with GNU Parallel (Tange, 2018).

### 2.5 Experimental setup

To estimate the material properties of the graft and stent, we performed uniaxial tensile strength measurements on an ElectroPuls E3000 fatigue testing machine (Instron, Norwood, MA) with standard methodology (Said et al., 2020). We imposed ten preconditioning cycles before obtaining the final measurements. Additionally, differential scanning calorimetry was performed to derive the phase transformation behavior of nitinol.

**FIGURE 3:**
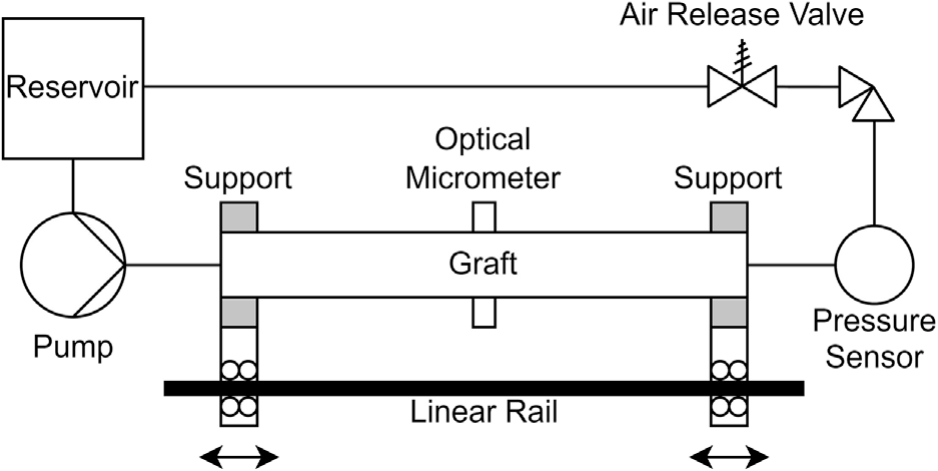
Schematic diagram of the experimental setup. The supports can be either locked in position or allowed to move independently on the linear rail.

The compliance of the assembled grafts was then measured in a hydraulic circuit, that was specifically built for this application (Figure 3). The circuit consisted of a pump with programmable volume flow rate, stiff tubing, a reservoir with controlled temperature and two rigid cylindrical supports. The supports were mounted separately on a linear guide, and the graft was secured on the supports with removable clamps. The linear guide allowed precise control of the distance between the supports, or equivalently of the grafts longitudinal prestretch. The temperature was either set to room temperature (21°C), or to body temperature (36°C) via heating of the reservoir, to investigate the effect of temperature on the properties of the grafts. To prevent liquid leakage across the graft wall, we inserted a thin-walled, waterproof latex tube inside the prosthesis. The latex tube had larger diameter than the graft, and its area compliance was at least three orders of magnitude higher than the graft’s compliance, when measured with the same methodology. The compliance of the circuit, including the valves and the sensors, was four orders of magnitude lower. Thus, the latex tube and the circuit would have negligible effect on the compliance measurement. Trapped air could increase the measured compliance, so we removed it from the circuit via an air relief valve and by circulating the liquid multiple times. At the mid-level of the graft, we placed a calibrated pressure transducer (ADInstruments, Dunedin, New Zealand). We used a Keyence LS-7030 optical micrometer (Keyence Corporation, Osaka, Japan) to measure the external diameter and the radial displacement, by aiming at the inner layer at the middle point of the graft.

The testing protocol consisted of ten preconditioning and ten experimental stress-controlled cycles of inflating the prosthesis in the 50–200 mmHg pressure range with a loading frequency of 0.2–0.5 Hz. The magnitude of the volume flow rate was kept constant throughout each experiment, resulting in non-linear loading due to the nature of the experiment, which consisted in inflating a cylindrical sample. The applied testing and measurement procedure was similar to the standard testing procedure for vascular prostheses (Ferrari et al., 2019). Using the same protocol, we tested both our CM grafts and the commercial, gold-standard grafts, that we used as the inner layer. The length of the tested samples was at minimum 15 cm and their diameter was 14–30 mm.

### 2.6 Data acquisition and processing

We recorded the signal from all sensors simultaneously on a PowerLab (ADInstruments, Dunedin, New Zealand) data acquisition device at 1 kHz. All measured signals were filtered using a low-pass filter with cut-off frequency of 50 Hz to remove the utility frequency and higher frequency noise. The sampling and cut-off frequencies were sufficiently higher than the inflation frequency. First, we used the measured pressure and the imposed volume flow rate of the programmable pump to create the pressure-volume curve. Since woven grafts have minimal axial rigidity, they bend and deviate from the centerline as the transmural pressure increases. We accounted for the pressure-induced bending by assuming that the graft will adopt the shape of a beam fixed at both ends under a bending load. Therefore, we derived analytically a function that provides the length of the bent graft based on the measured maximum radial deflection. With this function, we adjusted the volume of the graft so that it corresponded only to the radial inflation, without the change of volume caused by the elongation due to bending.

Based on the experimental measurements, we calculated the volume compliance as $C_{v}=dV/dP$ , and the area compliance $C_{a}=dA/dP$ , where V,A are the graft’s adjusted internal volume and lumen area, respectively, and P is the intraluminal pressure. Additionally, we defined distensibility $D_{a}$ as the area compliance normalized by the lumen area and we calculated it as $D_{a}=C_{a}/A_{ref}$ , where $A_{ref}$ is the lumen area at the corresponding pressure or at a reference pressure of 100 mmHg. Because the uncompressed PET graft had larger diameter than the combined graft, the area distensibility was more appropriate to perform comparisons between them. We, also, applied the same equations to calculate the aforementioned quantities based on the results of the FEM simulation. In the FEM case, however, we started the calculation from the mean radial displacement of the mesh nodes on the inner surface of the middle (lengthwise) section of the simulated part. Thus, we were able to avoid the boundary effects caused by the outermost stent struts.

### 2.7 Statistical methods

We programmed the optimization algorithm, the experimental data processing code and the statistical analysis in Matlab (MathWorks, Natick, Massachusetts). Means were compared with paired samples *t*-test or with Welch’s *t*-test as appropriate. Multiple comparisons were corrected with the Holm-Bonferroni procedure. Correlations were evaluated using linear regression analysis and the Pearson’s correlation coefficient (r). The level of statistical significance for all analyses was set to 0.05. Values are reported as mean (SD).

## 3 Results

### 3.1 Graft design

**FIGURE 4:**
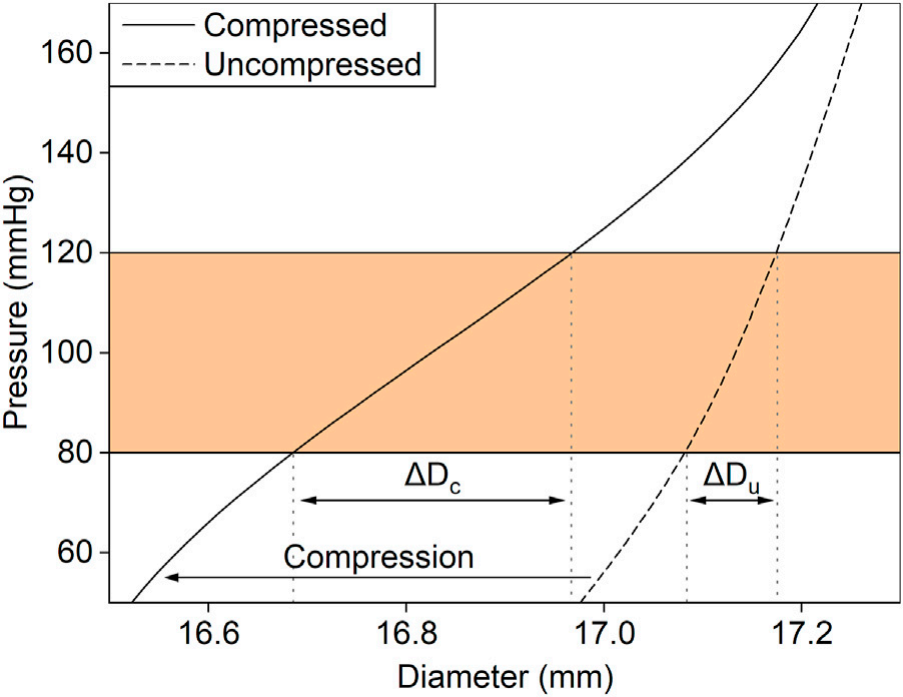
The effect of the radial compression with the metallic stents on the experimentally measured relationship between diameter and pressure of an aortic graft. Radial compression (arrow) results in a leftward shift of the pressure–diameter curve. For the same change in intraluminal pressure (shaded region), the change in diameter is larger in the compressed graft (ΔD_c_) than in the uncompressed (ΔD_u_).

The relationship between pressure and diameter of both CM and PET grafts was measured experimentally in the hydraulic circuit (Figure 4). The radial compression with the metallic stents was equivalent to a leftward shift of the pressure–diameter curve of the graft. By utilizing the minimal rigidity of the compressed inner layer, which under zero load was in a collapsed state, we were able to increase the change in diameter for the same change in pressure, resulting in an increase of the graft’s compliance. The maximum equivalent stress of the metallic stents was 380 MPa at 200 mmHg of pressure, which is within the elastic region of the material, while the maximum equivalent total strain was 0.02.

### 3.2 FEM simulation validation and optimization

**FIGURE 5:**
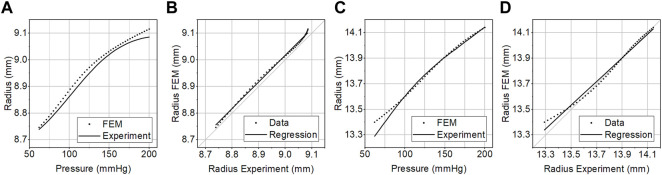
**(A, C)** The pressure–radius curve of the compliance-matching graft measured experimentally *in vitro* (Experiment) or predicted by the FEM simulation (FEM). **(B, D)** Linear regression analysis between the predicted and the experimental graft radius at 50–200 mmHg of internal pressure. The experimental curves were generated by averaging the curves of three samples of optimal **(A, B)**, and larger suboptimal **(C, D)** grafts.

**FIGURE 6:**
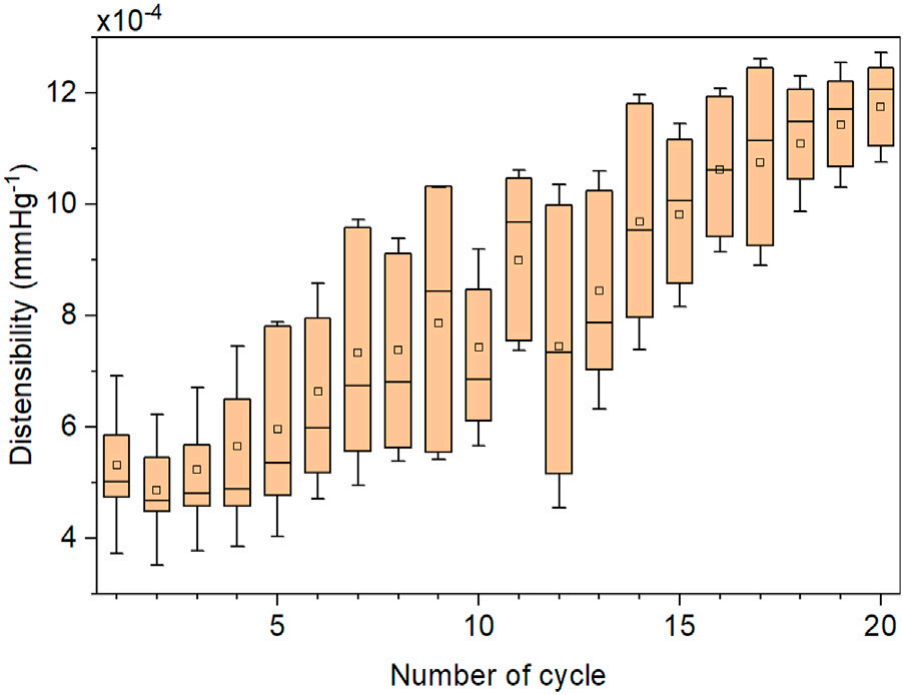
Representative evolution of the distensibility in consequent Box-Behnken designs (cycles) during a single optimization process. The optimization results were derived from the FEM simulation. Each cycle of the applied 6-factor Box-Behnken design consists of 49 FEM simulations.

We validated the FEM simulation against the experimental data. The FEM simulation estimated the internal radius of the prototyped CM grafts with a root-mean-square error (RMSE) of 0.022 mm over the physiological aortic pressure range (80–120 mmHg) and with a RMSE of 0.018 mm over the extended pressure range (50–200 mmHg). The RMSE in the estimation of area compliance was 0.018 mm^2^/mmHg and 0.025 mm^2^/mmHg for the same pressure ranges, respectively. Additionally, we assessed the accuracy of the FEM simulation with linear regression analysis (Figure 5). The slope of the regression line was *β* = 1.014, *p* < .001, and the two variables were strongly correlated, *r =* 0.99, *p* < .001. The optimization process proceeded rapidly (Figure 6), and the maximum distensibility was reached in all cases within less than 20 optimization cycles, while the computation time of each optimization cycle was less than 2 h in two 36-cores nodes.

### 3.3 Distensibility

**FIGURE 7:**
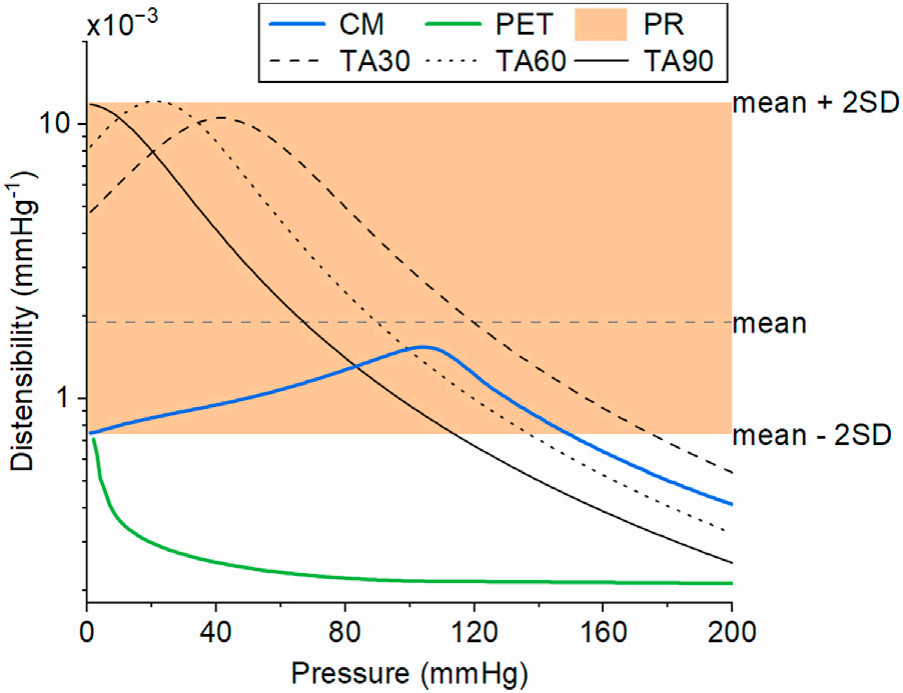
The distensibility of the CM graft (solid blue line), as measured *in vitro* by averaging three samples, is within the physiological distensibility range of healthy, normotensive adults (PR, shaded region) over a wide range of pressure. The distensibility of a standard PET graft (solid green line), as measured *in vitro*, is included for comparison. The PET graft was used as the inner layer of the CM graft. The data for the physiological distensibility range (PR) were derived from (The Reference Values for Arterial Stiffness’ Collaboration, 2010). The representative pressure-dependent distensibility of explanted thoracic aortas of people aged 30, 60, and 90 years (TA30, TA60, and TA90, respectively) were derived from (Langewouters et al., 1984). The CM and PET lines correspond to experimental data at 36°C and 10% longitudinal prestretch.

**FIGURE 8:**
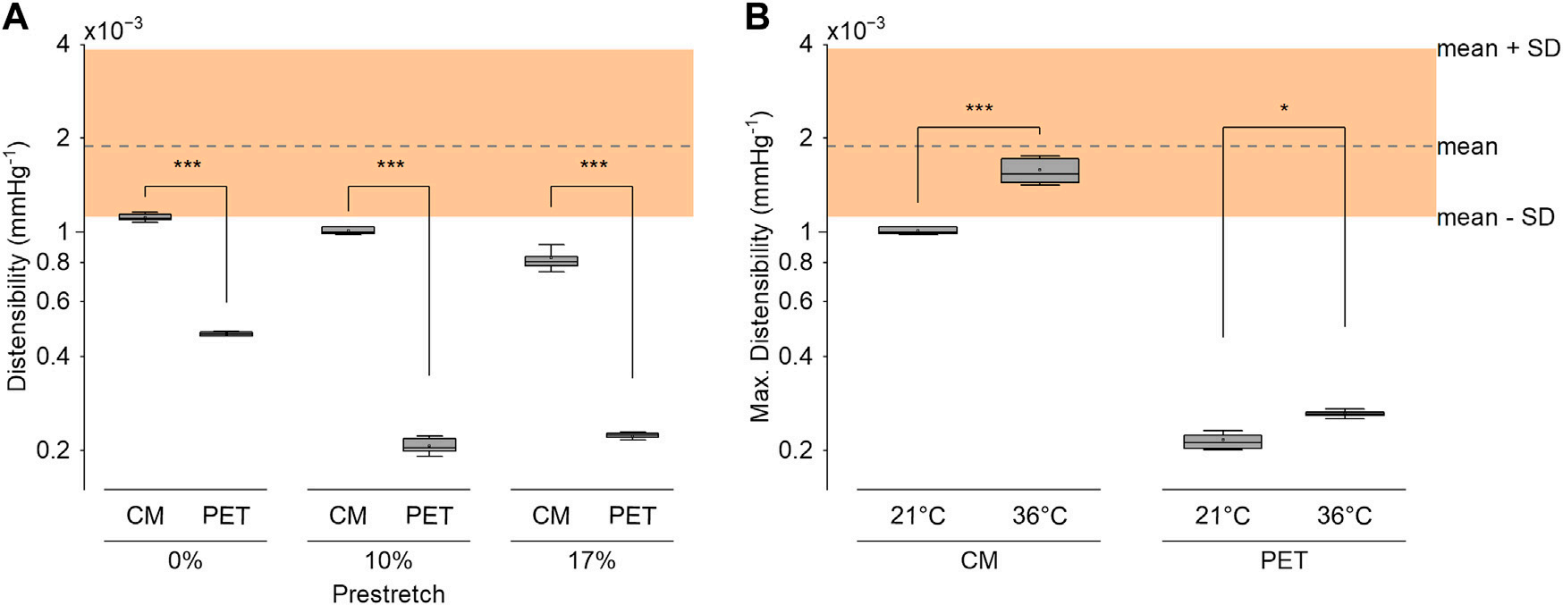
**(A)** The distensibility of the compliance-matching (CM) and the PET grafts at 3 levels of longitudinal prestretch at 21°C. **(B)** Maximum distensibility of the CM and PET grafts in the 80–140 mmHg pressure range at 21°C and 36°C at 10% longitudinal prestretch. The shaded region represents the reference distensibility range of healthy normotensive adults, as derived from (The Reference Values for Arterial Stiffness’ Collaboration, 2010). Three samples were tested in each condition with ten measurements per sample. *p < .05, ***p < .001.

A radial compression of 10%–15% was enough to increase the compliance without introducing excessive folding in the inner layer. The optimized CM grafts reached a distensibility of 1.60 × 10^−3^ mmHg^−1^ (*SD* 0.17 × 10^−3^) at a reference pressure of 100 mmHg under the expected implantation conditions (body temperature, 10% longitudinal prestretch). The CM graft distensibility was within the reference range of aortic distensibility of healthy, normotensive adults (The Reference Values for Arterial Stiffness’ Collaboration, 2010) up until 150 mmHg of pressure (Figure 7). Taking into account the pressure dependency of distensibility, the CM grafts were within the reported physiological range of healthy, human, ascending aortas (Langewouters et al., 1984). PET grafts were out of the physiological and the pressure-dependent ranges at all examined pressure values. The longitudinal prestretch significantly reduced (*p* < .001) the distensibility of both compliant and PET grafts (Figure 8A). The distensibility of compliant grafts was reduced by 9% (*p* < .001) when we introduced a 10% longitudinal prestretch, while the same prestretch caused a 56% reduction (*p* < .001) in the distensibility of PET grafts. At all tested levels of prestretch, the distensibility of CM grafts was higher than that of PET grafts (*p* < .001). An increase in temperature resulted in a significant increase (CM: *p* < .001, PET: *p* < .05) in the maximum distensibility of both graft types in the 80–140 mmHg pressure range (Figure 8B). However, CM grafts showed higher augmentation in maximum distensibility (57%) compared to PET grafts (21%) when the temperature was increased from environmental to body temperature. At both temperatures, CM grafts showed significantly higher maximum distensibility than PET grafts (*p* < .001).

## 4 Discussion

### 4.1 Compliance-matching

The results of our study suggest that our novel graft can reach the compliance of healthy human aortas, even that of young adults. Our CM graft could reach physiological levels of compliance for most of the aortic pressure range expected in adults. This accomplishment separates our CM graft from gold-standard aortic grafts, whose compliance is multiple times lower than the physiological range. The experiments presented here show that it is possible to increase and regulate the compliance of a graft by compressing it with optimally designed stents. To the best of our knowledge, the proposed prosthesis is the first that could solve the issue of compliance mismatch, utilizing a biocompatible design that could retain its properties after implantation, without sacrificing the remaining characteristics of existing gold-standard grafts. The CM graft compliance is within the reference values for aortic stiffness based on pulse wave velocity measurements (The Reference Values for Arterial Stiffness’ Collaboration, 2010), and within the aortic distensibility range measured by a recently proposed analysis method of magnetic resonance imaging (Cecelja et al., 2022). Furthermore, the compliant stent-graft design allows the implantation of the prosthesis both by open surgery and by endovascular procedures.

### 4.2 Comparison with stent-grafts

The concept of stent-grafts has already been introduced for endovascular aortic reconstruction. Contrary to the proposed solution, the stents are utilized in the existing prostheses only for applicability purposes to allow for endovascular repairs. In fact, the stents reduced the compliance of the prosthesis and increased the pulse wave velocity, a clinical measure of aortic stiffness, at the abdominal aorta (Morris et al., 2016). Existing stent-grafts also have an unequal distribution of elastic properties between stented and unstented regions (Singh et al., 2017), an issue that can lead to arterial wall damage (Greenberg et al., 2000). Arterial wall damage can also be caused by the oversizing technique (Karck and Kamiya, 2008), according to which stent-grafts with larger diameter than the vessel are selected, in an attempt to reduce compliance mismatch and to firmly secure the grafts in place. The influence of stents and the oversizing technique on local and global stiffening have been investigated in explanted aortic stent-grafts (Agrafiotis et al., 2023). The researchers found a significant loss of distensibility in the stented parts of the grafts, between 25% and 88%, compared to the unstented part. Additionally, the distensibility of the explanted stent grafts was as low as 1.7 × 10^−5^ mmHg^−1^, which is two orders of magnitude lower than the CM graft and the physiological range. These findings are contrary to the role of the stents in the CM graft, which is primarily to increase the compliance, although they could be used additionally for endovascular delivery. The endovascular delivery of the CM graft could be easily achieved by compressing the CM graft with a retractable sheath. Given that the nitinol stents are designed to be in their superelastic region at body temperature, the CM graft would behave as a self-expanding stent-graft, after the removal of the retractable sheath.

### 4.3 Effects of compression and decompression

We showed that it is possible to increase the compliance up to approximately six times by introducing radial compression. The compression was small enough to allow the prosthesis to function properly but without altering the implantation procedure compared to a standard graft. Due to the minimal rigidity of thin fiber-based tubes in the compressed state, the compression method is expected to work not only with PET but also with other types of synthetic and even biological grafts. The stent parameters would have to be re-optimized based on the specific material combination. The concept of compression has been investigated previously, utilizing springs and compression plates (Legerer et al., 2020), a solution that would be impossible to be implemented in an implantable graft. The researchers reported a reduction of 22% in the pulse wave velocity of the thoracic aorta after compressing the graft, which corresponds to an increase of 1.6 times in distensibility. This value is lower than the maximum achieved increase in distensibility caused by our graft (∼6 times). The difference could be attributed to the suboptimal design, and to the selection of the spring constants and the compression level.

The effect of the unfolding and gradual engagement of the compressed fibers of the inner layer can be clearly seen in the pressure-dependent behavior of the graft (Figures 5, 7). As expected because of the induced compression, the behavior of the graft is a non-monotonic function of pressure. As the pressure increases, the inner graft becomes unfolded and its fibers start to bear load, at pressure higher than 130 mmHg, thereby reducing the distensibility. This behavior matches closely to human arteries, whose non-monotonic behavior is defined by the collagen fibers that initially unfold and slowly begin to bear load as pressure increases (Langewouters et al., 1984). On the contrary, this behavior does not resemble the concave pressure diameter curve, and the monotonic loss of distensibility of dacron grafts as pressure increases.

### 4.4 Effects of implantation conditions

As shown, it is important for the prostheses to have the desired properties at the expected implantation conditions. That is because conditions, such as longitudinal prestretch and temperature, greatly affect the properties of the prostheses (Figure 8). Longitudinal prestretch is unavoidable during surgical implantation and it is additionally augmented by the periodic movement of the heart during the heart cycle, due to the extension of the aorta caused by the retraction of the aortic annulus (Plonek et al., 2018). The aortic annulus movement plays an important role in the volumetric compliance of the aorta (Pagoulatou et al., 2021). Our graft could retain its distensibility at an acceptable level even under reasonable longitudinal tension. This ability could be crucial in clinical applications. Longitudinal prestretch immediately caused a notable depletion of distensibility in gold-standard grafts. Other novel graft designs, such as the caterpillar-like graft (Singh and Wang, 2014), might suffer from significant reduction in compliance due to prestretch. In the absence of prestretch, that graft reached similar distensibility (1.5 × 10^−3^ mmHg^−1^) to our graft, but it quickly diminished to non-physiological levels with increasing pressure. The stented version of the same graft had even lower distensibility (0.5 × 10^−3^ mmHg^−1^). It is also expected that as the knitted fibers of that or of similar grafts extend in the axial direction, they could quickly lose their ability to extend circumferentially, thereby reducing their compliance further.

Another aspect of implantation conditions, that is often overlooked, is how the organism’s reaction to the prosthesis will affect its long-term properties. Woven dacron grafts, similar to the inner layer of the CM graft, were found to maintain their diameter after the implantation, showing only a 5.5% diameter increase after a mean period of 13 years (Troost et al., 2009). This was not the case for knitted or older types of dacron grafts and for grafts made from other materials (e.g., ePTFE) (Schroeder et al., 2009). The compliance of explanted grafts has not been thoroughly investigated, but evidence suggest that tissue growth reduces the compliance over time (Usui et al., 1988; Nagano et al., 2007). Moreover, PET grafts increased in diameter and lost up to 60% of their initial compliance when they were subjected to 24-h loading *in vitro*, while the effects were more prominent in larger grafts (Lucereau et al., 2020). The researchers attributed the loss of compliance to the irreversible rearrangement of yarns after loading. We expect that these finding will have only a moderate effect on our design, because most of the loading is distributed to the stent, while the inner layer remains mostly compressed without bearing load. Yarn rearrangement could therefore be reduced, and the stent could protect the graft from excessive dilation. The stent is also less prone to a change of properties due to tissue growth, at least compared to fiber-based grafts or novel designs with multiple lumina (Chen et al., 2012; Singh and Wang, 2014). The latter category of grafts could be very susceptible to infiltration of blood in the interluminal space with consequent coagulation, resulting in severely altered mechanical properties. Regarding the fatigue life of the stent, the stress and strain amplitudes at 80–120 mmHg were 60 MPa and 0.038%, respectively, and even for above normal pressure differences of 150 mmHg, did not exceed 80 MPa and 0.2%, respectively. Therefore, the stents operate well within the endurance limits of nitinol as evaluated by both stress-life and strain-life methods even at 200 mmHg of mean pressure (Robertson et al., 2012), while the deformations were within the elastic region of the material. This was an expected result given the relatively small load variations caused by the pulsatile pressure in the human cardiovascular system, and the high mechanical strength of nitinol.

### 4.5 Finite element modeling and optimization of grafts

The FEM simulation and the computational optimization aided and accelerated the development of the compliant grafts. We validated successfully the results of the FEM simulation using experimental data of the whole stent-graft (Figure 5). The FEM simulation was able to capture the behavior of the compressed inner layer with the surrounding stent, and it predicted the radius with enough precision to have an acceptable estimation of the pressure-dependent compliance. When we simulated only the stent layer of the graft, which reduced the model complexity, it proved to be insufficient to describe the behavior of the whole graft, and hence unsuitable for optimization. This could be attributed to the fact the stents should not be as compliant as possible, but they should have sufficient strength to keep the inner layer compressed within physiological pressure levels and allow it to expand only at higher pressure.

In the literature there is a notable absence of validated models that could estimate the pressure-dependent properties of vascular prostheses. We found FEM simulations that were constructed for fiber-based grafts and they produced very accurate results (Bustos et al., 2016; Amabili et al., 2018). Similarly, the properties of vascular stents have been investigated numerically (Jayendiran et al., 2020; Ong et al., 2022) and in some cases the models were validated with experimental results (Bernini et al., 2022). Nevertheless, in the case of stent-grafts, there are multiple finite element and fluid structure interaction models that have not been validated (Demanget et al., 2013; Jayendiran et al., 2018), whereas validation has only been performed for the stent stress during crimping (Zhou et al., 2019) or during balloon expansion (Kleinstreuer et al., 2008) and for the shape of bent or kinked stent-grafts (Demanget et al., 2012; Perrin et al., 2015).

As for the computational optimization, its use is still limited in the field of vascular prostheses and, especially, stent-grafts, in spite of the many advantages the method has to offer. Researchers have used computational fluid dynamics to find the optimal volume ratio and diameters of abdominal endoprostheses to reduce thrombosis risk (Polanczyk et al., 2017). Multi-layer tissue engineered grafts with small diameter have also been successfully optimized in terms of layer thickness to reach physiological compliance with simple FEM simulations (Tamimi et al., 2019; Furdella et al., 2021). The effective application of computational optimization in a more complex problem, as described in this work, could encourage researchers to implement similar methodologies in stent-graft design. An automated optimization method could also pave the way for patient-specific vascular prostheses, which optimally match the anatomy and compliance of each patient. For that the optimization process could be easily modified to yield stent-graft designs that match a pre-specified value of compliance, an approach that would be very beneficial especially for younger patients. Personalized polymer meshes, that match the aortic root anatomy, have been used for external aortic root support in Marfan syndrome patients with promising results (Treasure et al., 2014). An expansion of this technique could lead to a new generation of personalized aortic grafts with optimal geometrical and elastomechanical characteristics.

### 4.6 Limitations

Our findings are limited to the extent of validation that a combined *in vitro* and *in silico* approach allows. Future experiments in suitable animal models will be necessary to prove that the proposed graft could offer augmented compliance *in vivo*, and to verify if the augmented compliance translates to improved clinical outcomes and clinical significance. The animal experiments would also be appropriate to assess whether the addition of stents could lead to problems such as graft delamination or rupture, although based on existing stent-grafts, this is a rare complication. Furthermore, to reduce computational time we optimized and evaluated the compliance in quasi-static simulations and experiments, while the matching compliance was assessed by neglecting the effects of anisotropy, viscoelasticity, and prestress of the arterial wall. Additional optimization would be required to design CM grafts that could be compressed to the desired diameter for endovascular repairs, and anchor stents would be mandatory at the proximal and distal ends to secure the graft on the aorta.

## 5 Conclusion

We conclude that the proposed compliance-matching graft can attain the compliance of healthy, human aortas and it could mitigate the phenomenon of compliance mismatch along with the associated risks. The proposed grafts retain their compliance within physiological levels in a wide range of operating conditions, contrary to existing prostheses that are multiple times stiffer than the aorta. The selected materials are biocompatible, thus enabling the potential for *in vivo* experiments to prove the beneficial effects of increased graft compliance on the cardiovascular system. The methodology, that was developed in this study, not only enables the optimization of stent-graft properties, but it also unlocks the potential for personalized vascular prostheses in the future. We believe that the compliant grafts could reduce the post-operative complications that are caused by current prostheses, thereby improving the quality of life and life expectancy of patients submitted to aortic replacement surgery.

## Data Availability

The original contributions presented in the study are included in the article/Supplementary Material, further inquiries can be directed to the corresponding author.
