# Supplementary material for: Design and computational optimization of compliance-matching aortic grafts
Source: Front Bioeng Biotechnol. 2023 Jun 29;11:1179174. doi: 10.3389/fbioe.2023.1179174 (PMC10341153; doi:10.3389/fbioe.2023.1179174)
Supplement: Supplementary file 1 [file DataSheet1.PDF]

Supplementary Material

Design and computational optimization of compliance-matching aortic grafts

Georgios Rovas*, Vasiliki Bikia, Nikolaos Stergiopulos

*** Correspondence: Corresponding Author: georgios.rovas@epfl.ch**

1. Material characterization

Tensile tests used for the parameters of the material models of the finite element simulations.

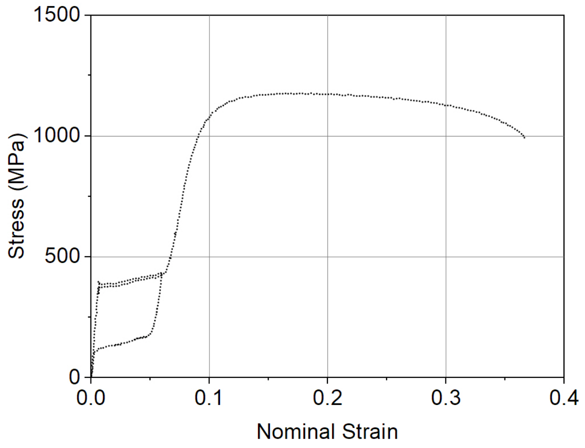

Supplementary Figure 1. Tensile test of Nickel Titanium.

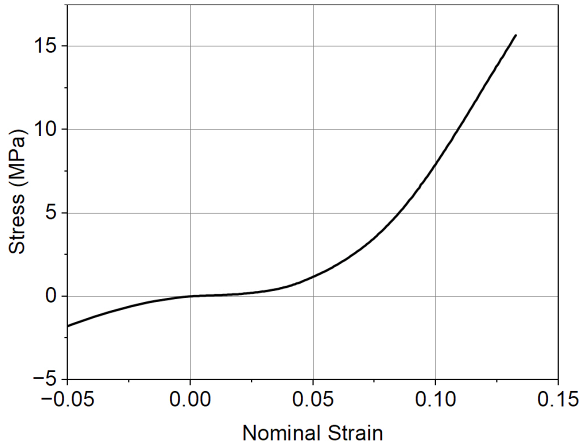

Supplementary Figure 2. Tensile test of the PET graft samples in the circumferential direction.

2. Nickel Titanium (Nitinol) material model

We used a user material routine (UMAT) following the shape-memory material model proposed by Auricchio and Taylor (Auricchio and Taylor, 1997). This theory is based on the concept of generalized plasticity and physical principles. In brief, the theory decomposes strain $\Delta\varepsilon$ into two parts: a purely linear elastic component $\Delta\varepsilon^{el}$ and a transformation component $\Delta\varepsilon^{tr}$ as $\Delta\varepsilon = \Delta\varepsilon^{el} + \Delta\varepsilon^{tr}$. The transformation component is calculated by the fraction of austenite that is transformed to twinned martensite and by a transformation potential. The temperature regulates the stress levels at which the transformation occurs, while a linear stress-temperature behavior is assumed, an assumption that is close to reality for small temperature changes. Additional information on the material model can be found in the original publication.

The parameters of this model were derived from the stress-strain curve (Sup. Fig. 1) and from differential scanning calorimetry. They are summarized in Supplementary Table 1. The maximum stress in all simulations was lower than the plastic region of the material. Therefore we chose not model the plastic behavior, which resulted in significantly reduced computational time.

Supplementary Table 1. Nickel Titanium material model parameters

Parameter	Value
Austenite elasticity	57 GPa
Austenite Poisson's ratio	0.33
Martensite elasticity	24 GPa
Martensite Poisson's ratio	0.33
Transformation strain	0.047
Loading stress-temperature gradient	6.7 MPa/K
Start of transformation loading	370 MPa
End of transformation loading	430 MPa
Reference temperature	19 °C
Unloading stress-temperature gradient	6.7 MPa/K
Start of transformation unloading	210 MPa
End of transformation unloading	110 MPa
Start of transformation stress (compression)	370 MPa
Volumetric transformation strain	0.047

3. Mesh independence

The results of the mesh independence study can be seen in Sup. Fig. 3. Based on this study, we selected the reported mesh sizes, not by the total number of elements, since the geometry changes in every simulation, but based on the number of elements per strut or graft cross-section. The number of nodes was approximately 30% higher than the number of elements for each case.

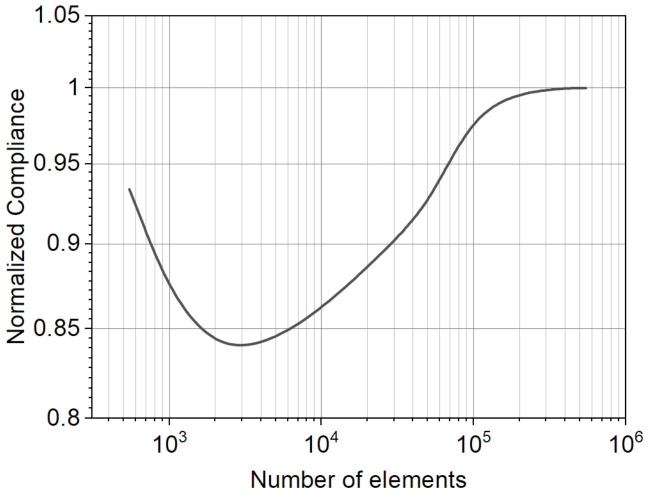

Supplementary Figure 3. Mesh independence study results on a representative geometry. Compliance was normalized with respect to the compliance of the simulation with the maximum number of elements.
